# Supplementary material for: Comparison of Efficacy of 2% Chlorhexidine Gluconate–Alcohol and 10% Povidone-Iodine–Alcohol against Catheter-Related Bloodstream Infections and Bacterial Colonization at Central Venous Catheter Insertion Sites: A Prospective, Single-Center, Open-Label, Crossover Study
Source: J Clin Med. 2022 Apr 17;11(8):2242. doi: 10.3390/jcm11082242 (PMC9031555; doi:10.3390/jcm11082242)
Supplement: Supplementary file 1 [file jcm-11-02242-s001.zip › jcm-1654438-supplementary.pdf]

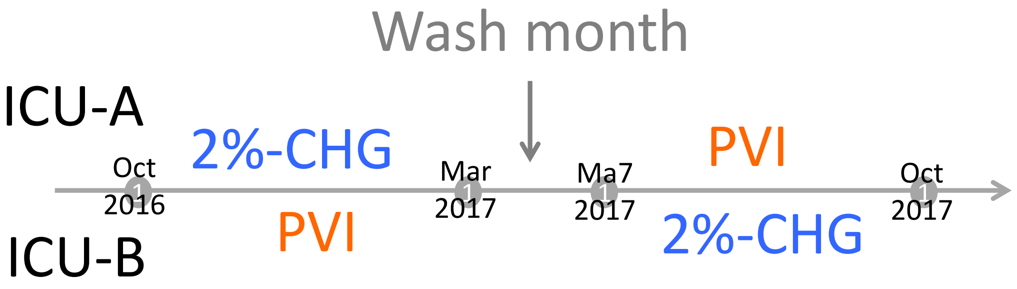

CHG=Chlorhexidine-alcohol
PVI=10% Povidone-iodine-alcohol

Figure S1. Flow chart of intervention. 2 ICUs were assigned to use 2% CHG-alcohol or 10% PVI-alcohol in the CVC care bundle. There were two intervention periods of six months separated by a washout period of one month

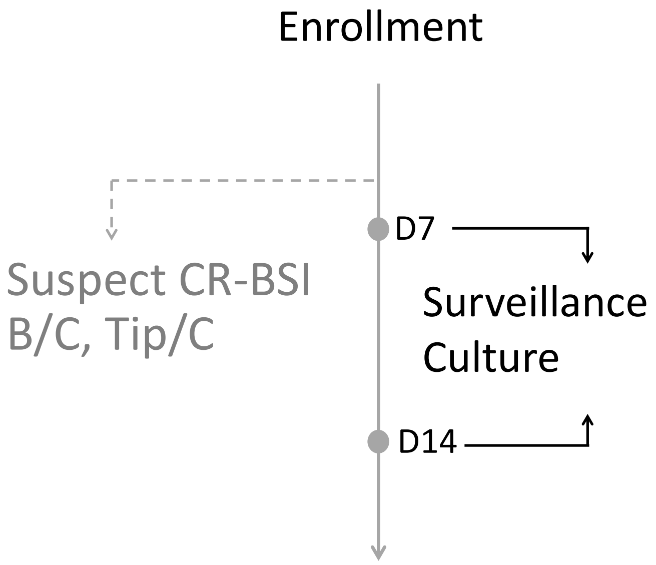

Figure S2. The surveillance culture over the CVC insertion site was done to evaluate the growth of skin flora on day 7 and day 14 after catheter insertion. Paired blood sample and tip of catheter were sent for aerobic culture whenever CRBSI was suspected. Abbreviations: CRBSI, catheter-related bloodstream infection; B/C: blood sample for aerobic culture; Tip/C: tip of catheter for aerobic culture.
